# Innovative Use of Gallic Acid as a Crosslinking Agent for Gelatin: A Biocompatible Strategy for 3D-Printed Scaffolds in Tissue Engineering

**DOI:** 10.3390/pharmaceutics17080951

**Published:** 2025-07-22

**Authors:** Teresa Carranza, Elias Hasan, Pedro Guerrero, Koro de la Caba, Ana Marina Ferreira

**Affiliations:** 1 BIOMAT Research Group, University of the Basque Country (UPV/EHU), Escuela de Ingeniería de Gipuzkoa, Europa Plaza 1, 20018 Donostia-San Sebastián, Spain; teresa.carranza@ehu.eus (T.C.); pedromanuel.guerrero@ehu.es (P.G.); 2 School of Engineering, Faculty of Science, Agriculture & Engineering, Newcastle University, Newcastle upon Tyne NE1 7RU, UK; elias.hasan@newcastle.ac.uk (E.H.); ana.ferreira-duarte@newcastle.ac.uk (A.M.F.); 3 BCMaterials, Basque Center for Materials, Applications and Nanostructures, UPV/EHU Science Park, 48940 Leioa, Spain; 4 Proteinmat Materials SL, Avenida de Tolosa 72, 20018 Donostia-San Sebastián, Spain

**Keywords:** gelatin, gallic acid, alkaline oxidation, 3D printing

## Abstract

**Background/Objectives:** Gallic acid, a natural phenolic compound, was used as a crosslinking agent to achieve protein–polyphenol conjugation under alkaline conditions, presenting an innovative approach to stabilize gelatin. **Methods:** The formulated inks were evaluated for their rheological properties and 3D printing performance. Once the scaffolds were printed, physicochemical properties were assessed by color changes and FTIR. Additionally, three different post-processing methods were studied to avoid toxicity: incubation in PBS, incubation in NaOH followed by PBS neutralization, and incubation in HCl followed by PBS neutralization. **Results:** The inks exhibited shear-thinning behavior with self-supporting capacity after extrusion, indicating their suitability for use as inks in 3D printing. After printing, changes in color and in the amide I band/amide II band ratio were observed due to alkaline oxidation, confirming the gelatin crosslinking. Among the tested treatments, incubation in PBS or NaOH followed by neutralizing with PBS proved to be the most suitable for obtaining cytocompatible scaffolds. The mechanical properties demonstrated the suitability of the proposed crosslinking systems for creating scaffolds. **Conclusions:** This strategy confirms that gallic acid-mediated crosslinking under alkaline conditions enables the fabrication of cytocompatible and mechanically stable gelatin-based scaffolds, making them suitable for tissue engineering.

## 1. Introduction

Gelatin, a protein of great significance in the biomedical field, has gained substantial interest owing to its biocompatible and biodegradable character in physiological environments [1]. Derived from the hydrolysis of collagen, a vital component of the extracellular matrix, gelatin exhibits a remarkable affinity with natural tissues, attributed in part to the arginine–glycine–aspartic (RGD) motifs that facilitate cellular adhesion to this macromolecule. However, its viability as a substitute, obtained through thermally induced gelation, is limited by its tendency to dissolve under physiological conditions [2,3,4].

Various strategies have been explored to stabilize this protein; among them, the use of compounds like glutaraldehyde or carbodiimides. However, these crosslinking agents, while effective and easy to handle, pose challenges for direct in situ gelation in vivo environments and/or maintaining cell growth after seeding due to their inherent toxicity [5]. Another alternative under research is the use of bio-based approaches such as phenolic compounds.

Gallic acid is one of the most interesting phenolic compounds due to its applications in the pharmaceutical industry, both in in vivo and in vitro studies [6,7]. This molecule has demonstrated antifungal and antibacterial properties, as well as cytotoxic effects against cancer cells. Furthermore, it possesses antioxidant properties that shield human cells from oxidative stress [8]. Moreover, gallic acid has demonstrated that it can crosslink proteins at basic pH, inducing protein–polyphenol conjugates and, thus, being an interesting phenolic compound to stabilize gelatin [9,10,11,12].

Strategies involving gelatin blends with gallic acid have been developed to create food films with antimicrobial properties, resulting in enhanced surface microstructure and improved thermal stability of the films [13,14]. Modified gallic acid has also demonstrated the ability to crosslink gelatin while maintaining antibacterial activity. This modification enhances the mechanical and UV barrier properties of the fabricated structures [15]. The use of gallic acid with gelatin has shown improvements in the adhesive properties of blends used as coatings for bio-based films like PLA. These coatings enhance the oxygen barrier properties and exhibit sensitivity to oxidation in the covered food products [16]. In recent years, it has also been discovered that both UV light and pH have significant effects on the properties of hydrogels based on gallic acid and gelatin [17].

While multiple methods have been explored for employing gallic acid/gelatin systems in film production for packaging purposes, there is still a lot to be researched on the use of these systems in the field of 3D printing for tissue engineering applications. This fabrication technique is especially interesting for achieving geometries with complex internal patterns, which need to be porous enough to allow nutrients and water to pass through but still be mechanically strong enough to withstand bending or tensile stress [18]. Until now, the literature has focused on forming covalent interactions between gallol groups and polymeric matrices, followed by crosslinking using different strategies such as physical mechanisms like heat or metal ions or oxidizing compounds like NaIO_4_ [9,19,20]. However, little research has been conducted on the use of gallic acid to physically crosslink polymeric matrices, and even less on utilizing this system in additive manufacturing, and even less on using alkali conditions to crosslink the gelatin polymer. One possible reason for this lack of literature may be the toxicity associated with the oxidation products of gallic acid when oxidized under alkaline conditions. This effect has been recently reported in the literature by Guyot et al., where they linked the cytotoxicity of catechol-containing materials to the release of quinones formed through the oxidation process [21]. Quinones are derivatives of aromatic compounds formed by converting an even number of –CH= groups into –C=O groups, with any necessary rearrangement of double bonds, resulting in a fully conjugated cyclic dione structure. It has been demonstrated that quinones can form through oxidation under alkaline conditions in compounds such as gallic acid [8,21].

In this work, gallic acid/gelatin inks were formulated and printed to ensure the internal complexity of the scaffold, which was crosslinked by oxidation under alkaline conditions. This method simplifies the ink preparation process, eliminating the need for pre-conjugated polymers to achieve crosslinking. These scaffolds were subjected to different post-processing methods to ensure the cytocompatibility of the materials, thereby overcoming the cytotoxicity barrier previously reported in the literature.

## 2. Materials and Methods

### 2.1. Materials

Porcine gelatin (250 bloom) was purchased from Sancho de Borja, Spain. Gallic acid (≥98.0% purity) was obtained from Merck, Madrid, Spain. NaOH and PBS (phosphate buffer saline) were supplied by Sigma, Madrid, Spain.

### 2.2. Ink Formulation

A 15% (*w*/*v*) gelatin was dissolved in water and mixed with 1.5% (*w*/*v*) gallic acid at 80 °C for 30 min under magnetic stirring. Once dissolved, the pH was adjusted to 10, and the ink was immediately transferred into 10 mL syringes to prevent contact with oxygen.

### 2.3. Rheological Analysis

Thermo Scientific Haake RheoStress1 rheometer (IFI, Vigo, Spain), equipped with a Peltier-controlled system and a 35 mm diameter with a 2° cone plate geometry sensor, was used for the ink characterization.

First, the linear viscoelastic region was determined by a shear stress sweep, and a shear stress of 0.2 Pa was selected for the following rotational tests. A temperature sweep from 34 °C to 10 °C at 1 Hz was performed to determine the proper printing temperatures. A frequency sweep was carried out from 0.01 Hz to 100 Hz at 29 °C (based on the printing experiences), and a flow test was performed from 0.01 s^−1^ to 1000 s^−1^ at the same temperature.

Finally, the structure recovery test was performed using three different intervals: the first, at 0.01 s^−1^, simulating the material inside the syringe; the second, at 30 s^−1^, simulating extrusion; and the third, at 0.01 s^−1^, to simulate the material deposition onto the printing platform. The temperature selected was 29 °C in the first and second intervals to simulate the temperature inside the syringe, while 20 °C was selected in the third interval to mimic the platform temperature.

### 2.4. Scaffold Manufacturing

A scaffold consisting of a 15 mm diameter and 3 mm height cylinder was designed using Solid Edge (Siemens, Munich, Germany). The slicing to obtain the gcode was performed using Cura 5.4.1 slicing software (Ultimaker, Utrecht, The Netherlands) using the following printing parameters: 0.4 mm (22 G) diameter needle, 0.15 mm layer height, 0.4 mm layer width, 10 mm/s printing speed, 29 °C printing head temperature, and 20 °C printing platform temperature. Finally, the gcode was transferred to the 3D printer to obtain the scaffolds fabricated using a domoBIO 2A printer (Domotek, Donostia-San Sebastian, Spain) equipped with a refrigerated platform and a heated syringe head.

### 2.5. Crosslinking

#### 2.5.1. Colorimetric Analysis

CR-400 Minolta Chroma Meter colorimeter (Konika Minolta, Madrid, Spain) was used to obtain colorimetric data through the CIELAB color scale (L* = 0 (black) to L* = 100 (white), −a* (greenness) to +a* (redness), and −b* (blueness) to + b* (yellowness)) and, thus, to obtain the oxidation kinetics of gelatin/gallic acid ink. The total color difference, ΔE*n, was calculated by Equation (1), using the measurement at time zero as reference [22]. To measure the difference between current (*n*) and previous (*n* − 1) measurements, dE*n was defined as expressed in Equation (2).

(1) $${∆E^{*}}_{n}=\sqrt{({∆L^{*}}_{n}{)}^{2}+({∆a^{*}}_{n}{)}^{2}+({∆b^{*}}_{n}{)}^{2}}$$

(2) $${dE^{*}}_{n}={∆E^{*}}_{n}-{∆E^{*}}_{\left(n-1\right)}$$

#### 2.5.2. Fourier Transform Infrared (FTIR) Spectroscopy

FTIR spectroscopy was performed using Bruker ALPHA II FTIR spectrometer (Madrid, Spain), equipped with an attenuated total reflectance (ATR) crystal (ZnSe). A total of 32 scans at 4 cm^−1^ resolution were recorded from 4000 to 750 cm^−1^ wavenumbers at different times.

### 2.6. Scaffold Characterization

#### 2.6.1. Swelling Analysis

First, scaffolds, dried in a climatic chamber, were weighed (*W*_0_) and then submerged in a phosphate-buffered saline (PBS, 0.01 M and pH 7.4) solution for varying time intervals. After immersion, the wet samples were carefully dried with filter paper to eliminate excess liquid and re-weighed (*W_t_*). The swelling percentage was calculated using Equation (3):

(3) $$Swelling\left(\%\right)=\frac{W_{t}-W_{0}}{W_{0}}$$

#### 2.6.2. Mechanical Testing

TA.XT plusC Texture Analyzer (Aname Instrumentación Científica, Madrid, Spain) was used to measure the mechanical properties of the scaffolds, and the Exponent Connect 8.0.11.0 Lite software (Stable Micro Systems, Godalming, UK) was used to process the data. The test was conducted with a load cell of 5 kg and a crosshead rate of 5 mm/min, and five samples were conducted for each system.

#### 2.6.3. Biological Characterization

##### Sample Washing

Prior to biological characterization, the samples were washed using three different methods: acid incubation (with HCl 0.1 N), alkaline incubation (using NaOH 0.1 M), and neutral incubation using PBS buffer (pH 7.4), as reported by other authors [21]. The samples were stirred for 72 h in the three different media at 7 mg sample/mL media concentration, and after this, they were neutralized with PBS (same concentration) for 96 h to seed cells. During this process, and to analyze the leaching molecules, the media were aliquoted and analyzed using UV–Vis spectroscopy. A wavelength scan ranging from 220 to 850 nm was performed using a FLUOstar^®^ Omega plate reader and MARS Data Analysis Software V5.02 R3 was used to process the data (BMG Labtech, Ortenberg, Germany). Aliquoted samples (200 µL each) were labeled according to the method applied (PBS, HCl, or NaOH; incubation or neutralization; and the corresponding time). Cleaning media were used as blanks to subtract the background.

##### Sample Sterilization

After the incubation and cleaning processes, the samples were sterilized by immersion in ethanol for 30 min, and after being washed with PBS twice, they were placed in 24-well plates and exposed to 254 nm light for 90 min.

##### Cell Culture

Adult human dermal fibroblasts (C-12302), PromoCell, Heidelberg, Germany), at a density of 25,000 cells per well, were incubated at 37 °C in a humidified incubator with 5% CO_2_ to perform biocompatibility assessments over different times. Fibroblasts were passaged weekly upon reaching confluence, collected using 0.25% Trypsin-EDTA, and centrifuged at 1200 rpm for 5 min at room temperature. The resulting cell pellet was resuspended in the culture medium to create a uniform cell suspension. Dulbecco’s Modified Eagle’s Medium (DMEM) supplemented with 10% (*v*/*v*) inactivated fetal bovine serum (FBS) and 1% (*v*/*v*) penicillin–streptomycin was used as media.

##### Indirect and Direct Cytotoxicity Assays

Two types of tests were performed: an indirect cytotoxicity assay, where pre-adhered cells on the well plate were exposed to the DMEM medium previously incubated with the samples subjected to different cleaning methods (PBS, HCl, and NaOH), and a direct seeding assay, where cells were cultured directly on top of the scaffolds treated in various ways. In the indirect toxicity assay, cells cultured with untreated DMEM served as a positive control and were considered 100% viable at day 1. To determine the cytocompatibility of the samples, the scaffolds were incubated with cells. The constructs (scaffolds with cells) were subsequently removed to characterize, ensuring that only cells adhered to the scaffolds were counted, rather than those remaining in the well. This approach was applied for both viability tests and different staining assays.

##### Cell Counting Kit-8 (CCK-8)

Cell viability was measured using cell counting kit-8 (CCK-8) (Sigma, London, UK). According to the manufacturer’s protocol, the reagent was used at a concentration of 1/10 in cell media. It was incubated for 2 h at 37 °C and 5% CO_2_ and the results were seen using FLUOstar^®^ Omega plate reader (BMG Labtech, Ortenberg, Germany), recording the absorbance at a 450 nm wavelength.

##### Live/Dead^®^ Cell Viability Assay

A Live/Dead^®^ cell viability assay (Thermo Fisher, Runcorn, UK) was used over different time points to monitor the cell status in the indirect and direct cytotoxicity and cytocompatibility experiments. Calcein AM and ethidium homodimer-1 were added to final concentrations of 1:2000 and 1:1000, respectively, with 500 µL of the mixture added to each well.

##### DAPI/Phalloidin Morphological Assay

DAPI (NucBlue) and phalloidin (ActinRed 555) dyes (Invitrogen, Paisley, UK) were used to perform the morphological analysis of fixed cells. Cells were fixed using 4% (*w*/*v*) PFA for 10 min, and afterward, they were washed three times with PBS. Then the cells were permeabilized using Tween-20 for 5 min. After another three washings, two drops of Actin Red-555 per mL of PBS were used for 30 min to mark F-actin. Finally, after removing the Actin red-555, two drops of NucBlue (DAPI) per PBS mL were used for 5 min to mark the nucleus.

Live/dead and morphological assays were observed in an Evos M5000 (Invitrogen, UK) microscope. For direct cytocompatibility tests, samples were transferred onto a slide to avoid imaging cells attached to the well plate.

### 2.7. Statistical Analysis

The SPSS Statistics 25.0 software program (IBM, Armonk, NY, USA) was used to perform the statistical analysis. A one-way analysis of variance (ANOVA) with Tukey’s multiple comparisons post hoc test was used with the level of significance established at *p* ≤ 0.001.

## 3. Results and Discussion

### 3.1. Rheological Analysis of the Inks

3D printing inks must be extrudable through the printing nozzle and maintain their shape once deposited on the printing platform [23], and thus, the study of the elastic behavior of the material at the temperatures in both the printing nozzle and the printing platform is necessary [24].

**Figure 1:**
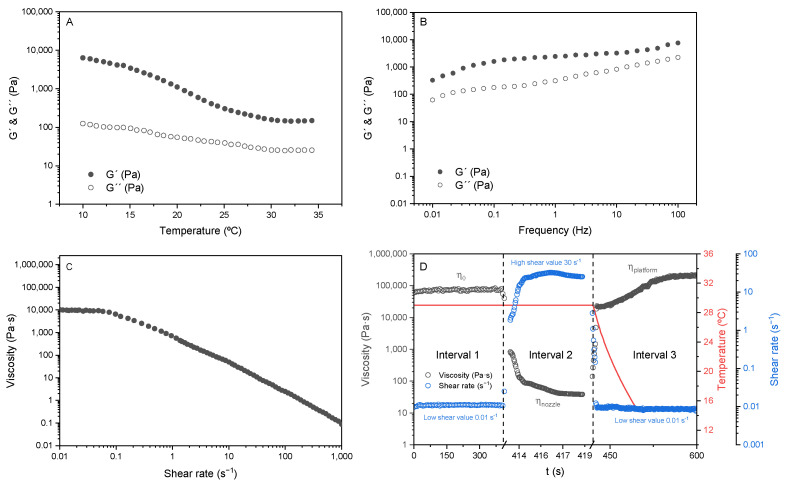
(**A**) Temperature sweep, (**B**) frequency sweep, (**C**) flow test, and (**D**) recovery test for 3D printing ink.

The temperature sweep of gelatin/gallic acid ink performed from 10 °C to 35 °C is shown in Figure 1A. As can be seen, there is a big difference between storage (G’) and loss (G’’) moduli up to 15 °C, indicating the solid-like behavior. As the temperature rises from 15 °C, this difference starts to decrease up to 28 °C, when the difference is maintained up to the end of the temperature sweep. Frequency sweep is represented in Figure 1B, where G’ goes over G’’ during all the sweep, indicating the solid-like behavior of the ink and a good self-supporting ability, favorable for 3D printing applications. To ensure the ability of the ink to be extruded, a flow test was carried out and is shown in Figure 1C. As can be seen, the viscosity decreases as the shear rate increases, indicating a shear-thinning behavior. Additionally, a plateau was observed until 0.1 s^−1^, indicating that the ink needs a minimum shear rate to start flowing. Finally, the recovery test (Figure 1D) simulates the shear rate applied during three different stages: the first simulates the material in the syringe, being the shear near zero; the second simulates the shear applied on the nozzle wall during a printing stage; and the third simulates the material deposition on the platform with almost no shear applied. The temperature also varies from 29 °C in the first and second stages to 15 °C in the last stage. As can be seen, the material recovers more than 100% of its original viscosity once the shear is removed, demonstrating the capacity to maintain its shape once extruded, without showing breaks in its polymeric chain, which would cause the viscosity drop after the ink deposition.

The flowability of gelatin at 29 °C is influenced by its sensitivity to temperature, which facilitates chain movement through intermolecular hydrogen bonding; otherwise, the differences at lower temperatures can be explained by the presence of the triple helix [25]. These findings indicate that the inks are printable at 29 °C and remain stable on the platform at 15 °C. Additionally, the ink exhibited excellent recovery capabilities, attributed to the formation of hydrogen bonds at lower temperatures (simulating the platform) and the absence of chain breakages due to the shear applied.

### 3.2. Crosslinking

The effect of oxidation time was assessed by color and FTIR analyses. The oxidation of gallic acid can be monitored by the color change of the samples from yellow/brown to dark brown, as previously reported by other authors [26,27]. The colorimetric values of the printable ink, as a function of oxidation time, are shown in Table 1, where the values of L*, a*, and b* tend to be black, green, and blue, respectively. According to Grobelna and colleagues [28], the color change (dE* > 3.5) is noticeable to the human eye from 0 to 5 min, while the color change (1 < dE* < 3.5) from 10 min to 50 min is only noticeable by an experienced observer. From 50 min onwards, the color change (dE* < 1) is not noticeable, so the crosslinking cannot be monitored only by a color change, and FTIR spectroscopy was used.

**Figure 2:**
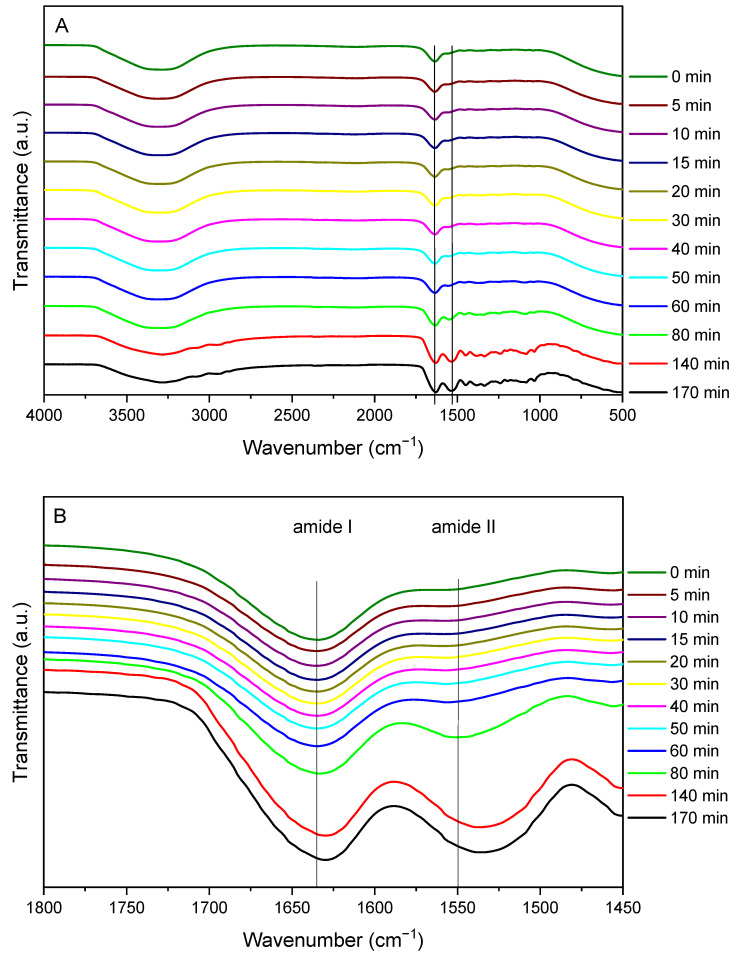
(**A**) FTIR spectra of gallic acid/gelatin inks as a function of oxidation time. (**B**) Spectra magnification from 1800 to 1450 cm^−1^.

FTIR spectra of the same sample at different oxidation times are shown in Figure 2A. The O-H and C=O stretching-related band of amide A is observed at 3325 cm^−1^, the C=O stretching of amide I at 1635 cm^−1^, and the amide II, related to N-H bending, at 1557 cm^−1^. As can be seen in Figure 2B, the amide I band is shifted as a function of time up to 1629 cm^−1^. In addition, a displacement of the amide II band was observed from 1557 cm^−1^ to 1537 cm^−1^.

The ratio between the areas of amide I and amide II at different times can be related to the crosslinking degree, as previously reported using lactose to crosslink gelatin-based films [29]. As observed in Table 2, the decrease in this ratio indicates that new interactions between the protein and the gallic acid were occurring. These interactions could be non-covalent, such as hydrogen bonding between the carbonyl and amino groups, or covalent through the quinone formed by the alkaline oxidation of gallic acid with nucleophilic amino acid residues (cysteine, lysine, methionine, and tryptophan) of gelatin [10], which may indicate that a crosslinking process is occurring. Under alkaline conditions (pH 10), gallic acid exists in equilibrium between the di-anionic and tri-anionic semiquinone radical forms [30]. These negatively charged species can interact physically with the polymeric matrix. Moreover, in the presence of oxygen, semiquinone radicals undergo auto-oxidation to form reactive o-quinones, which can react with protein nucleophiles via Michael addition [9]. Considering these results, samples were 3D printed and left to oxidize for two days.

The results reported in the literature on the use of gallic acid in tissue engineering have focused on the modification by click chemistry and subsequent crosslinking by metal coordination complexes and/or physical interactions. These processes require intermediate molecules such as EDC (1-ethyl-3-(3-dimethylaminopropyl)carbodiimide) and NHS (N-hydroxysuccinimide), among others, to incorporate the gallic acid into the polymeric chain, and dialysis cleaning to remove the intermediates from the hydrogel formed [17,19]. However, the process carried out in this work allows crosslinking in a single process, being a much simpler and more economical process to scale up.

### 3.3. Characterization of the Scaffold

**Figure 3:**
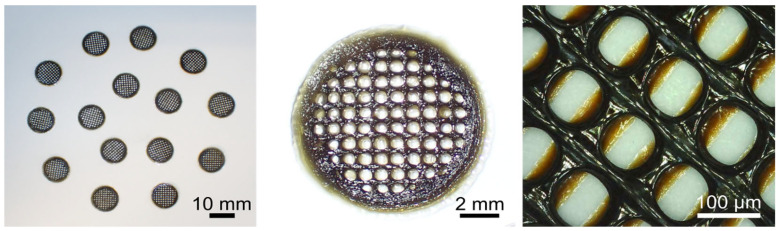
Images of the oxidized 3D-printed scaffolds at different magnifications.

Samples (n = 120) were printed and left to oxidize at room temperature and relative humidity of 70% for 48 h to be completely dehydrated (Figure 3). The ink remained printable after overnight storage at 4 °C, showing no signs of premature crosslinking. Moreover, during extrusion, no nozzle obstruction was observed, indicating that crosslinking occurs primarily after deposition on the printing platform. The scaffolds showed high homogeneity in porosity, with pores of 78.8 ± 7.5 µm, and in weight (49.8 ± 8.5 mg). Although there is some build-up of material at the junction points between different printing lines, this effect does not cause the structure to collapse. Therefore, it can be deduced that the ink becomes a strong gel once it is deposited on the printing platform, resulting in the high shape fidelity of the 3D-printed scaffold.

Scaffolds must possess sufficient mechanical stability to withstand external forces while allowing for cellular infiltration and nutrient exchange [18]. This makes the analysis of tensile properties and liquid medium uptake capacity necessary.

**Figure 4:**
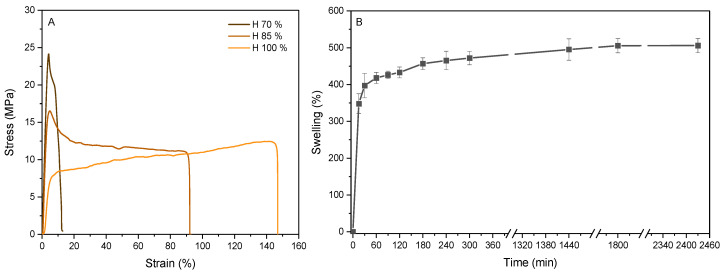
(**A**) Stress–strain curves of the scaffolds conditioned at different humidity levels and (**B**) swelling of crosslinked gallic acid/gelatin scaffolds.

The stress–strain curves (Figure 4A) illustrate the mechanical performance of the scaffolds conditioned at 70, 85, and 100% relative humidity. Various humidity levels were analyzed to determine the behavior of the scaffolds under the different humidity conditions present in the human body [31]. Elastic behavior is observed up to a deformation of 4–5%, indicating that the material has reached the yield point. However, the yield stress varies with the humidity, with the sample conditioned at the lowest humidity demonstrating the highest yield stress. Beyond this point, the material undergoes plastic deformation, making the deformation irreversible. Notably, while samples conditioned at 70% and 85% relative humidity exhibit a peak (yield point) followed by a gradual decrease in stress beyond the yield point, the samples conditioned at 100% relative humidity do not show a peak but rather an increase in stress beyond the yield point. The breaking point also depends on the humidity level, with the most humid samples breaking at higher strain percentages. These results suggested that water acts as a plasticizer, as also observed by other authors [32]. This effect is interesting when manufacturing a scaffold with tunable properties as a function of the degree of humidity since it could tailor the cellular response depending on the stiffness of the sample [33].

The optimal swelling of hydrogels is achieved when there is a balance between osmotic driving forces and cohesive forces. While the osmotic driving forces allow water or biological fluids to enter the hydrophilic matrix, the cohesive forces exerted by the polymer network counteract the expansion of the hydrogel, preventing the polymers from dissolving in the water. The extent of these forces depends especially on the crosslink density of the hydrogel; the higher the degree of crosslinking, the lower the swelling and, thus, also the degradation [18]. The swelling capacity is shown in Figure 4B, where the swelling reaches almost 500% after 40 h (2440 min). A sharp rise is noted in the first 15 min, indicating that the system is hydrophilic, but this increase slows down from 400% to 500% from 60 to 2440 min due to crosslinking. These values are lower than those obtained in the literature [34], demonstrating the crosslinking of gelatin with gallic acid at alkaline conditions in the presence of oxygen.

### 3.4. Biological Performance of the Scaffolds

**Figure 5:**
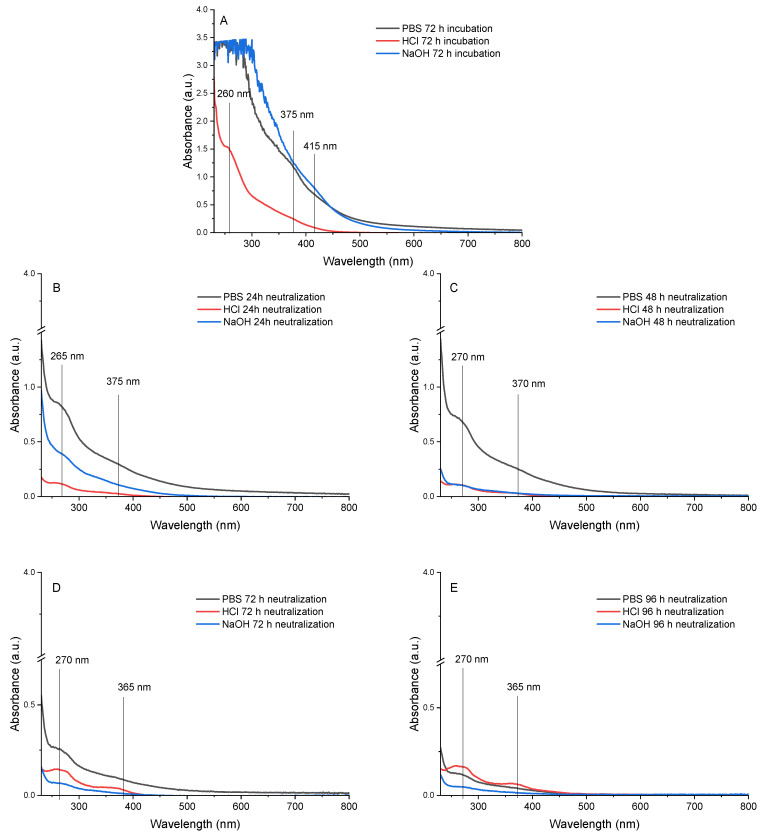
(**A**) UV–Vis spectra of incubation medium (PBS, HCl, and NaOH) and neutralizing medium (named after the incubation media used) at different time points: (**B**) 24 h, (**C**) 48 h, (**D**) 72 h, and (**E**) 96 h.

To ensure that the printed scaffolds were safe for cell growth, they were incubated with different solvents for 72 h: PBS (pH 7.4), HCl 0.1 M, and NaOH 0.1 M. Following this, the samples were stabilized in PBS for an additional 96 h, with the neutralizing media being changed once daily. A UV–Vis scan was performed to monitor the molecules leached in the incubation and neutralizing medium. Figure 5A–E show the UV–Vis spectra of leaching molecules in aliquoted incubation and neutralizing media at different time points. As shown in Figure 5A, a broad peak around 250–300 nm for PBS- and NaOH-incubation media, which overlaps the one at 375 and 415 nm for PBS and NaOH, respectively, was observed. For the HCl medium, one main asymmetric peak could be observed at 260 nm and an overlap from 300 nm onwards. It is noteworthy that, although there are no peaks, the absorbance values of the HCl incubation medium drop to zero starting at 450 nm, whereas in the other two methods, this does not occur until 700–800 nm. Once the incubation media were removed and the samples were stabilized with PBS (Figure 5B–E), two main peaks were observed for all the samples, one at 260–270 nm and the other one at 365–375 nm. Until 72 h of neutralizing (Figure 5B–D), the samples incubated with PBS exhibited the highest absorbance values, indicating a higher concentration of leached molecules in the neutralizing medium. However, the trend over time showed a decrease in absorbance, suggesting a gradual reduction in the release of these molecules. The same effect was observed over neutralizing times in the samples treated with NaOH, where any clear peak could be observed at 96 h neutralizing. The neutralizing media that were incubated with HCl had the opposite trend, in which the sharpest peaks with higher absorbances were observed at 72 and 96 h (Figure 5D,E).

The results obtained suggest that part of the gallic acid is released into both the incubation and neutralizing media, with an absorbance observed at 260–270 nm. These results have been previously reported in the literature, where it is noted that gallic acid exhibits two main absorbance peaks, one at 240 nm and another one around 280 nm. These peaks can vary slightly depending on the protonation level of the hydroxyl groups present on the aromatic ring [35,36]. The other peak observed around 370 and 415 nm could be related to the oxidation products, the gallate radical and the quinone that could be released [21,36,37]. Finally, the absorbance values observed up to 400 nm could be related to the semiquinones formed because of the basic pH value (around 500 nm) [21].

When the physical interaction between gelatin and gallic acid occurs, they can interact through hydrogen bonds, electrostatic interactions, or hydrophobic interactions [10]. The use of NaOH changes the polarity of the gallic acid molecules that have not covalently interacted with gelatin. These molecules can exist as gallic acid, gallate anion, or unreacted quinone, among other forms [38]. However, when the pH is adjusted with NaOH, this variability is reduced to gallate anion or quinone, which are less likely to interact via hydrogen bonds or electrostatic forces, thereby promoting their faster release as small molecules. Although the release level appears to be higher with PBS, as indicated by greater absorbance values, it is necessary to evaluate whether the samples exhibit cytotoxicity or not. The results indicate that PBS facilitates the release of non-reacted gallic acid, including non-reacted quinones. In this study, HCl was used as a control measure, since it is expected to show higher cytotoxicity in samples incubated with acid compared to those exposed to neutral or basic conditions because of a lower leaching observed, as shown by lower absorbance values.

**Figure 6:**
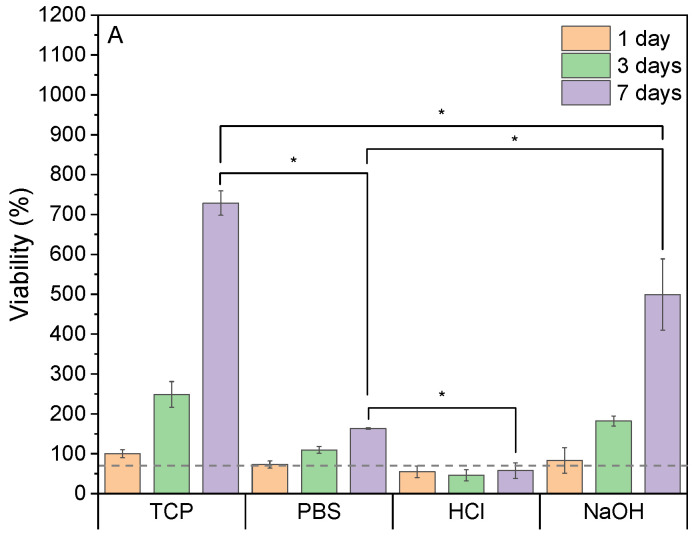

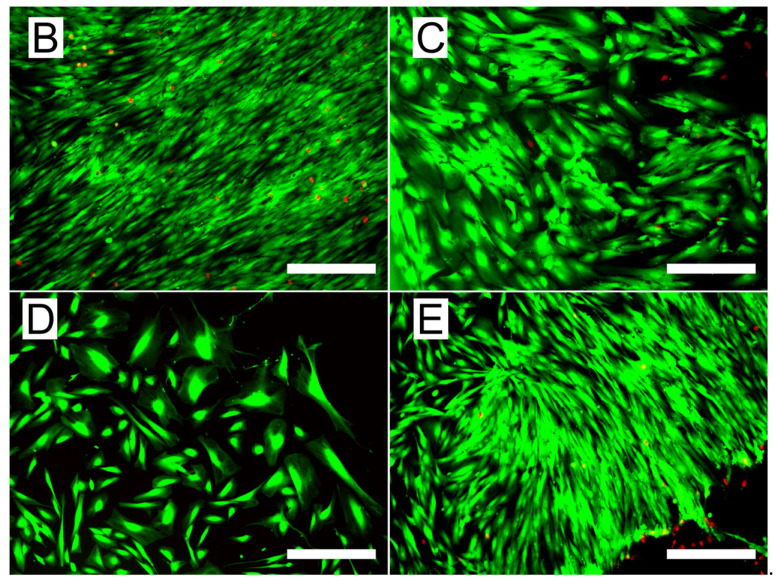
(**A**) Cell activity assessment through the exposition of the different leaching media to adult HDF cells after 1, 3, and 7 days and live/dead images (live cells = green, dead cells = red) of 7-day cultures of cells in (**B**) TCP-, (**C**) PBS-, (**D**) HCl-, and (**E**) NaOH-incubated sample mediums (scale bar 300 µm). Bars represent the mean values, while the lines with caps represent the standard deviations. Samples marked with whiskers are significantly different (* *p* ≤ 0.001) through Tukey’s range test (n = 3).

To assess the potential cytotoxic effect of samples and further leaching, cells were initially pre-seeded in tissue culture wells for 24 h to allow their attachment, followed by cell culture media change to be further incubated with the substrate previously in contact with the sample (leaching media). The samples were incubated with DMEM medium to obtain leaching media, named according to the method used to incubate them (PBS, HCl, or NaOH). Cells seeded in a tissue culture plate (TCP) and fed with fresh DMEM were used as the control group. Figure 6A shows the metabolic activity of fibroblasts at different time points in the presence of the different leaching media. Cells treated with PBS and NaOH leaching media showed over 70% viability, showing no cytotoxicity of these materials’ leaching media as per no statistically significant differences with the control TCP. Both methods showed an increasing metabolic activity and cell growth during days 3 and 7 of the culture; particularly the cells exposed to NaOH leaching media showed the highest growth. These results are confirmed by live/dead staining images at day 7 shown in Figure 6B–E, where cells incubated with HCl leaching media (Figure 6E) show the lowest and least confluent amount of viable cells.

These cell viability results are consistent with chemical analysis performed on the incubation media previously shown in Figure 5, where samples incubated with PBS and NaOH and subsequently neutralized with PBS suggest the highest levels of release and removal of unreacted gallic acid and quinones, promoting the highest viability values. This confirms that the issue of sample toxicity is being successfully addressed with these incubation treatments. Although this test demonstrates that the incubation treatments are effective in removing cytotoxic residues and by-products and therefore enhance the samples cytocompatibility (as per tests in ISO 10993), further studies of the behavior of cells in direct contact with the samples should be performed to assess cells interaction and attachment to the sample [39].

**Figure 7:**
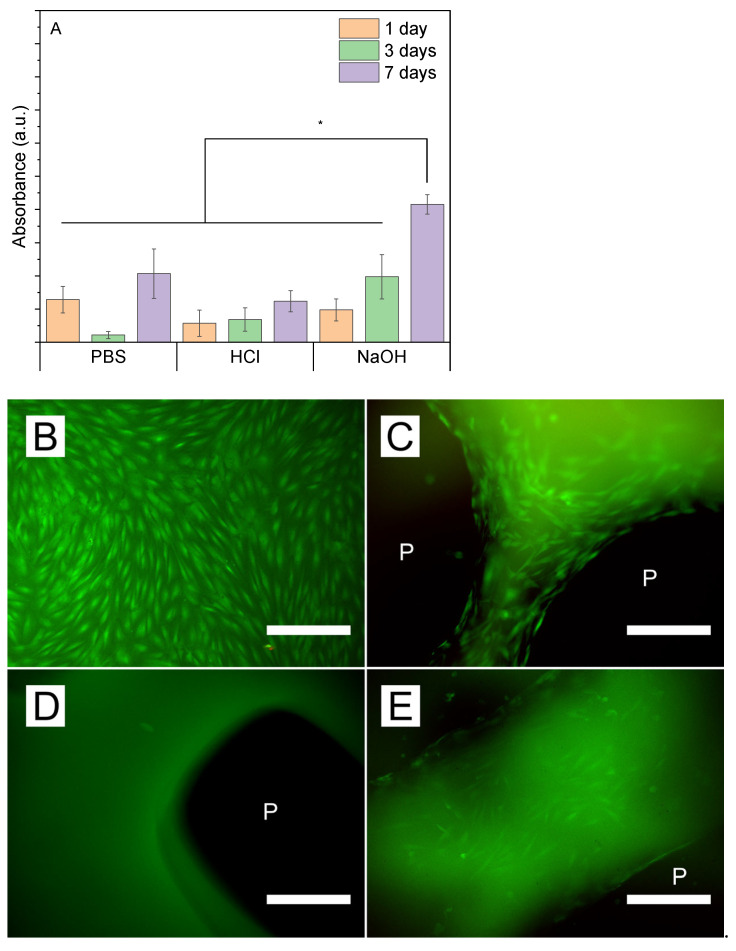
(**A**) Cell activity assessment of adult HDF attached to differently treated samples after 1, 3, and 7 days and live/dead images of 7-day cultures of cells in (**B**) TCP-, (**C**) PBS-, (**D**) HCl-, and (**E**) NaOH-treated samples (scale bar 300 µm; P, scaffold’s pores). Bars represent the mean values, while the lines with caps represent the standard deviations. Samples marked with whiskers are significantly different (* *p* ≤ 0.001) through Tukey’s range test (n = 3).

Figure 7A shows the HDF cellular viability at 1, 3, and 7 days when in direct contact with differently treated samples. Similarly to previously shown cytotoxicity tests (Figure 6), NaOH-treated sample condition shows the highest cell viability at the day 7 sample, confirming the cytocompatibility of the samples and their suitability for cell attachment and growth over time. Indeed, live/dead fluorescence images (Figure 7B–E) show the cellular viability (cells stained green) and their preference for spreading over the edges of the samples, with the highest cell confluency around the pores (P). Figure 7C,E show how the cells tend to accommodate and align at the edge of the pores, presenting similar viability to those grown onto the TCP (Figure 7B). In contrast, the samples incubated in HCl (Figure 7D) did not show confluency of live cells adhering to the surface, evidencing that samples incubated in PBS and NaOH present an improved cytocompatibility.

**Figure 8:**
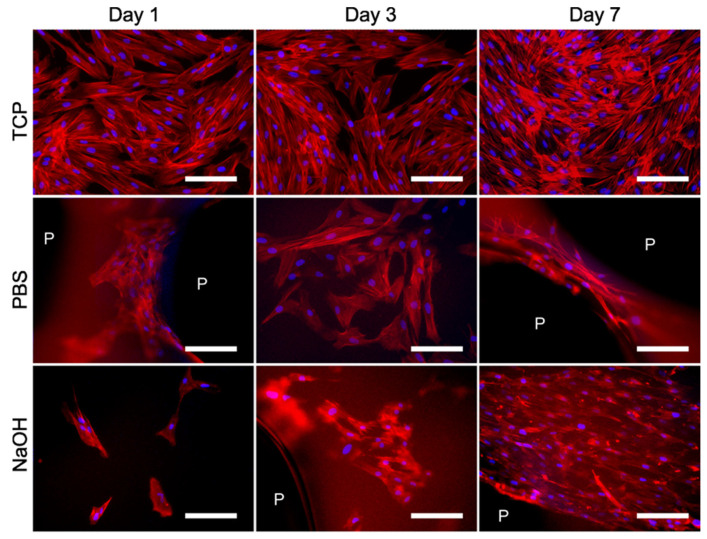
Human dermal fibroblast cell morphology (phalloidin, stained in red = actin filaments; DAPI, stained in blue = cell nucleus) at different days for TCP and for the samples incubated on PBS and NaOH (scale bar = 150 µm; P, scaffold’s pores).

To evaluate the morphology of HDFs, cells were observed on different culture days for their adherent cell phenotypes in samples incubated with PBS and NaOH (Figure 8). Cells seeded on TCP served as the positive control. All images reveal that the cells exhibit a typical fibroblast phenotype, characterized by a spindle-shaped elongated cytoplasm and a centrally located nucleus. Cells seeded in PBS showed their filopodia or cytoplasmatic extensions (actin filaments stained red in Figure 8) already on day 1, showing their cell–substrate and cell–cell interaction. Albuschies and Vogel demonstrated that filopodia plays a most significant role in topography sensing linked to the ability responsible for probing substrate stiffness [33,40]. Filopodia-mediated sensing provides an effective mechanism for durotaxis (a tendency of individual cells to follow stiffness gradients) and possibly other responses to the physical properties of the substrate by allowing cells to determine physical characteristics before moving over an area [33]. Interconnected fibers resist cell displacement much better. This is potentially significant in many physiological processes, as physically or enzymatically cleaved fibers are present in wound sites, whereas networks of interconnected ECM (extracellular matrix) fibers predominate in healthy tissues [40]. However, it is of interest when designing, e.g., a cellular dressing, as it could be designed in vitro, leading to rapid proliferation and then insertion into the wound site. This is also observed for the NaOH samples at day 3, although a higher confluency is observed for the NaOH samples compared to the PBS one. Indeed, this confirms the results obtained on metabolic activity of direct cytocompatibility assessment observed previously in Figure 7.

As observed previously in the live/dead images, a higher cell confluence is found around the pores. Moreover, for PBS and NaOH samples at day 7 (Figure 8), the cells appeared highly aligned and elongated in sample pore (P) filament. This cellular organization and orientation are not observed in the TCP, indicating that the construct properties, including stiffness, pore geometry, and material stretching formed during the 3D printing process, promote cellular organization and alignment of the HDF cells actin filaments.

The system studied proposes the use of printed structures based on gelatin and gallic acid capable of forming cytocompatible scaffolds not reported so far in the literature. Compared to traditional crosslinkers such as glutaraldehyde, genipin, or EDC/NHS, gallic acid offers several advantages, particularly in terms of biocompatibility. While glutaraldehyde is widely used for its strong crosslinking capacity, its cytotoxicity limits its biomedical applications due to the exposure to aldehydes [41]. Genipin, though less toxic, requires longer reaction times [42]. EDC/NHS provides efficient coupling for carboxyl and amine groups but may require strict control of reaction conditions [43]. In contrast, gallic acid allows mild, biocompatible crosslinking, as demonstrated in this study.

In addition to being scalable, the process presents adequate cytocompatibility and in vitro properties capable of ensuring the adhesion and growth of human fibroblast cells. The cell morphology analysis and the mechanical characterization results indicate that the scaffolds could be potentially used in biomedical applications such as a dermal substitute, as the cells could easily migrate in the 3D structure, interacting with the surrounding healthy tissue and the dermal substitute. Some authors have analyzed the effect of UV-C-irradiated gallic acid on its antimicrobial activity against Gram-negative bacteria [44]. According to these studies, gallic acid holds potential for the treatment and prevention of gastrointestinal diseases through its interaction with the gut microbiome and modulation of the immune response. Current in vitro assays and animal model results support the pharmacological and therapeutic interventions of gallic acid [45]. However, the exact mechanisms by which gallic acid acts on the gut microbiome and immune system remain unclear, and the effects of crosslinked systems containing oxidized gallic acid on immune response are even less understood. Still, investigations on the safety and viability of the scaffolds in vivo systems are required, as well as the interaction and response of the immune system to this scaffold material.

## 4. Conclusions

The formulated gallic acid/gelatin inks exhibited solid-like behavior and good self-supporting ability, making them highly suitable for 3D printing applications. Their shear-thinning properties allowed for smooth extrusion, while the working temperatures, 28 °C for extrusion and 15 °C for the printing platform, ensured compatibility with standard conditions. The recovery test demonstrated the ink’s ability to maintain its shape after extrusion, without showing polymeric chain breaks that could lead to a drop in viscosity, ensuring high stability during and after printing. The crosslinking process was effectively monitored through colorimetric analysis and FTIR, confirming the occurrence of oxidation. The 3D-printed scaffolds displayed high shape fidelity, retaining their designed geometries with precision. Water uptake studies revealed that the crosslinking process affected the swelling properties of the scaffolds, although they maintained sufficient water retention capacity, a critical feature for tissue engineering applications. To address the cytotoxicity associated with the crosslinking process, post-treatment with PBS and NaOH was tested. NaOH proved most effective, resulting in the highest cell confluence in both direct and indirect assays. These results confirm the potential of gallic acid–gelatin systems to produce cytocompatible scaffolds suitable for tissue engineering applications. Further work will be carried out to demonstrate the potential application of this system in vivo, with immune response being one of the key points. Additionally, long-term in vitro characterization, including degradation studies under physiological conditions, will be necessary to fully assess the performance and stability of the scaffolds over time.

## Figures and Tables

**Table 1:** L*, a*, b*, ΔE*, and dE* values of gallic acid/gelatin ink as a function of oxidation time.

tn (min)	L*	a*	b*	ΔE*	dE*
0	29.87 ± 2.20	−2.43 ± 0.43	20.48 ± 0.72		
5	19.78 ± 0.00	−2.75 ± 1.00	7.39 ± 2.00	14.88 ± 3.00	14.88
10	18.28 ± 1.01	−2.32 ± 0.79	5.59 ± 1.38	17.23 ± 1.53	2.50
15	16.72 ± 0.85	−1.60 ± 0.52	3.49 ± 0.69	19.84 ± 0.83	2.72
20	16.04 ± 0.83	−1.08 ± 0.45	2.50 ± 0.44	21.07 ± 0.55	1.32
30	15.36 ± 0.96	−0.44 ± 0.31	1.45 ± 0.36	22.36 ± 0.53	1.41
40	14.79 ± 0.79	−0.23 ± 0.10	1.06 ± 0.03	23.02 ± 0.49	1.20
50	14.63 ± 0.83	−0.14 ± 0.14	0.83 ± 0.09	23.30 ± 0.46	0.60
60	14.53 ± 0.83	−0.17 ± 0.16	0.80 ± 0.08	23.38 ± 0.50	0.12
80	14.91 ± 0.97	−0.05 ± 0.14	0.68 ± 0.11	23.27 ± 0.56	0.50
140	14.50 ± 0.65	−0.03 ± 0.09	0.69 ± 0.04	23.50 ± 0.38	0.44

**Table 2:** Amide I/amide II ratio as a function of time.

Crosslinking time (min)	0	5	10	15	20	30	40	50	60	80	140	170
Amide I/II	5.20	5.04	5.02	4.92	4.97	4.94	4.81	4.54	4.27	2.53	1.50	1.49

## Data Availability

The data is contained within the article.
